# Challenges in Drug Discovery for Neurofibromatosis Type 1-Associated Low-Grade Glioma

**DOI:** 10.3389/fonc.2016.00259

**Published:** 2016-12-20

**Authors:** Cora A. Ricker, Yuan Pan, David H. Gutmann, Charles Keller

**Affiliations:** ^1^Children’s Cancer Therapy Development Institute, Beaverton, OR, USA; ^2^Washington University School of Medicine, St. Louis, MO, USA

**Keywords:** pilocytic astrocytoma, neurofibromatosis type 1, drug therapy, low-grade glioma, pediatric neuro-oncology

## Abstract

Neurofibromatosis type 1 (NF1) is an autosomal dominant disorder that results from germline mutations of the *NF1* gene, creating a predisposition to low-grade gliomas (LGGs; pilocytic astrocytoma) in young children. Insufficient data and resources represent major challenges to identifying the best possible drug therapies for children with this tumor. Herein, we summarize the currently available cell lines, genetically engineered mouse models, and therapeutic targets for these LGGs. Conspicuously absent are human tumor-derived cell lines or patient-derived xenograft models for NF1-LGG. New collaborative initiatives between patients and their families, research groups, and pharmaceutical companies are needed to create transformative resources and broaden the knowledge base relevant to identifying cooperating genetic drivers and possible drug therapeutics for this common pediatric brain tumor.

## Lay Summary

Neurofibromatosis type 1 (NF1) predisposes children to the low-grade glioma (LGG), pilocytic astrocytoma (PA). While these brain tumors are slow growing, the locations in which PAs arise make them difficult to surgically excise – often devastating to a child‘s sight or neurological function. Furthermore, no cell lines or xenograft models exist from which to develop new targeted therapies, leading to a sole reliance on transgenic models for mechanistic insights. Increased collaborations with affected patients and their families may hold the key to building the necessary resources to unravel biology and new therapies for this cancer.

## Introduction

Low-grade gliomas account for 30% of primary pediatric central nervous system tumors, with PA predominating in children younger than 15 years of age (1). As a group, LGGs encompass both World Health Organization (WHO) grade I and II gliomas (2). Histologically, these tumors have low proliferative indices (<4%) with rare or absent mitotic figures and no evidence of necrosis. While distinguishing between these two malignancy grades can be challenging in some pediatric LGGs, PAs, unlike their WHO II counterparts, have characteristic eosinophilic granular bodies and Rosenthal fibers. In addition, PAs often contain a cystic component, especially when occurring in the cerebellum, and frequently harbor areas of compacted bipolar cells alternating with loose-textured multipolar cells and microcysts. Many PAs may also enhance, especially in their peripheral rim if a cystic component exists. Enhancement does not denote high-grade glioma (HGG) in this case. Also, intense meningeal enhancement may occur in the absence of a cystic component. Cystic components do not usually exist in optic nerve gliomas, but do exist often in PAs. Similar to other glial malignancies, these pediatric LGGs are immunopositive for expression of glial fibrillary acidic protein (GFAP) and Olig2. Finally, these tumors harbor a rich extracellular matrix with prominent infiltration of monocytes.

In general, children with PA can be divided into distinct subgroups based on their molecular etiologies. First, the majority of children with LGGs lack a genetic predisposition to brain cancer (sporadic PA). Pioneering genomic sequencing efforts by the Pfister laboratory and colleagues identified that the majority of sporadic PA tumors are caused by a somatic genomic rearrangement of the *KIAA1549* and *BRAF* genes to result in a fusion protein containing an unregulated and active *BRAF* kinase domain (3, 4). *BRAF^V600E^* mutations are most likely found in the extracerebellar and diencephalic regions (2). Subsequent studies revealed that this signature genomic alteration predominates in cerebellar and optic pathway PAs (5, 6). In addition to the *KIAA1549:BRAF* fusion, mutations in the fibroblast growth factor receptor 1 (*FGFR1*) and neurotrophic tyrosine kinase receptor 2 (*NTRK2*) genes are (more) common in non-cerebellar PAs (7).

The second group of children with PA includes those who harbor a germline mutation in the Neurofibromin (*NF1*) tumor suppressor gene and, therefore, have NF1 as the genetic etiology for their brain tumors. Examination of these tumors reveals somatic loss of the remaining *NF1* allele, resulting in bi-allelic *NF1* inactivation (8). Laboratory-based research over the past 20 years has revealed several potential opportunities for targeted inhibition of the growth control pathways deregulated in sporadic and NF1-associated PAs. The *NF1* gene encodes neurofibromin, a protein that primarily functions as a negative regulator of the RAS proto-oncogene. In this regard, loss of *NF1* gene expression leads to increased RAS activation and hyperactivation of the downstream RAS effectors, including the RAF/MEK/ERK and the PI3K/AKT (4) pathways, thereafter converging on the mechanistic target of rapamycin (mTOR) complex (9). Similarly, the *KIAA1549:BRAF* mutation results in increased MEK activation (10), which also operates to control cell growth through the mTOR complex (11). While less is known about the downstream signaling pathways operative in *FGFR1*- and *NTRK2*-mutant PAs, these receptor tyrosine kinase molecules are also known to activate RAS and RAS downstream signaling and regulate growth in numerous other cancers (12–16). These insights have resulted in the execution of early-phase clinical trials of MEK and mTOR inhibitors (NCT02285439 and NCT01734512; http://clinicaltrials.org), yet it is clear that additional resources and research will be required in order to develop personalized and effective therapies for these brain tumors.

Clinically, children with cerebellar PA come to medical attention when they present with signs and symptoms that reflect raised intracranial pressure, such as headaches, nausea/vomiting, blurred vision, and balance problems. Children with PAs involving the brainstem may exhibit reduced appetite, cranial neuropathies, and drowsiness, while those with optic pathway gliomas (OPGs) may have reduced visual acuity or early-onset puberty when affecting the hypothalamus. For this reason, accurate age-appropriate visual assessments are critical, especially for children with NF1, who are most at risk for the development of OPGs.

**Figure 1:**
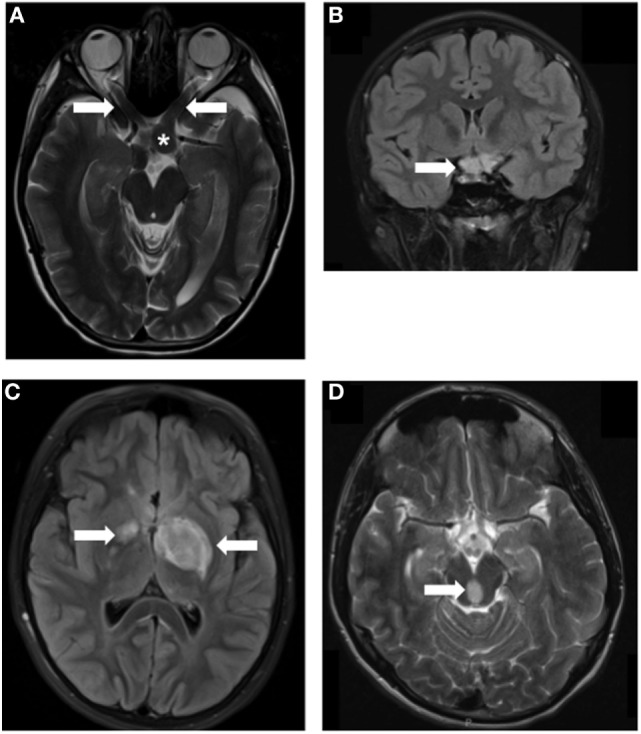
**MRI images of Neurofibromatosis type 1 (NF1)-associated low-grade glioma (LGG)**. LGGs (arrows) developing in children with NF1 at various locations, including optic nerves **(A)**, asterisk indicates the chiasm, chiasm **(B)**, thalamus **(C)**, and brainstem **(D)**. Arrows denote the location of the tumors.

Therapeutically, individuals with sporadic PAs typically undergo surgical resection of the tumor, when feasible. This approach is most often employed for those tumors located within the cerebellum, but not for midline or optic pathway tumors. In these situations, chemotherapy (carboplatin/vincristine) is employed to halt further tumor growth. However, allergic reactions have been reported from 2 to 30% of children with LGG (17). Risk of an allergic reaction from carboplatin and increased peripheral neuropathy from vincristine has led to vincristine being the first- or second-line monotherapy of choice (18–20). Since NF1-associated PAs are most commonly located in the optic pathway (optic nerve, chiasm, tracts, and radiations, Figure 1), surgery is rarely performed, and most children with symptomatic tumors are treated with chemotherapy in the absence of a tissue diagnosis unless patients demonstrate atypical symptoms and a biopsy is deemed unnecessary. Alkylator-based chemotherapy is typically avoided due to the risk of treatment-induced malignancy. Importantly, radiation therapy is rarely ever used due to the elevated risk of radiation-induced vasculopathy (21). With carboplatin/vincristine combination therapy, 56% (44/78) children with LGG exhibit an objective response, with two-year progression-free survival rates approaching 80% (22). In another study examining the combination of vincristine and carboplatin, 52% (12/23) of children with recurrent LGG had an objective response, and seven of these children showed tumor reductions greater than 50% (23). Although outcomes are far from optimal, approximately one-third of patients who have received chemotherapy demonstrated improvement of vision (almost all being carboplatin-based within the study). These responses speak to the pressing need to develop targeted therapeutic agents for these tumors that inhibit the specific growth pathways deregulated in NF1-associated tumors.

A total of 15–20% of children with NF1 develop LGGs (24–26), affecting 1:2,500 individuals worldwide. Over two-thirds of these PAs arise in the optic pathway (optic nerves, chiasm, tracts, and radiations), with another 15% located in the brainstem (27). Although rare, NF1 is one of the most common genetic disorders in humans, and PAs, when included in the LGG category, are the most common pediatric brain tumors. Thus, developing therapies for NF1-associated PAs could have a major impact on LGGs.

## Current Enabling Resources

### Patient Registries and Biobanks

Patient registries provide important information regarding demographic and clinical characteristics. Currently, there are several registries for brain tumors and others, specifically for NF1. Dr. Michael Fisher at the Children‘s Hospital of Philadelphia leads a large international study group for NF1-OPG aimed at defining the natural history and outcomes for these tumors. In 2012, the Children‘s Tumor Foundation (CTF) created the NF Registry. The NF Registry is a tissue repository managed by Precision Bioservices and curated by Patients Crossroads Inc. The NF registry notifies patients of new clinical trials and possible treatments, allows patients to compare medical histories, and enables patients to connect with researchers on pressing issues.

Additionally, the Washington University Neurofibromatosis (NF) Center created (May 2011) and currently hosts the Neurofibromatosis Type 1 (NF1) Patient Registry Initiative (28). This epidemiologic database serves a different purpose than the CTF Registry. It was specifically designed to facilitate clinical association studies that can be mechanistically explored in the laboratory. As such, this registry has been invaluable for assessing how internet-based epidemiological research can be optimally conducted (29, 30) and for identifying unanticipated associations between specific clinical features for future risk assessment analysis (31–33).

Although not specific to NF1, the National Cancer Data Base is a program established by the American College of Surgeons in collaboration with the American Cancer Society to provide oncology results from Commission on Cancer (CoC)-accredited hospital registries. In addition, the Central Brain Tumor Registry of the United States provides an invaluable resource for descriptive statistical data on all primary tumors, while the Swedish Cancer Registry houses similar data on LGG and HGG (34). Several other cancer registries, which track LGGs from around the world, are summarized in Table 1.

**Table 1:** **International Cancer Registries for Low-Grade Astrocytoma**.

Cancer registry	Location
French Registry of Children Solid Tumors https://epidemiologie-france.aviesan.fr/fr/epidemiologie-france/accueil	France
STEP Registry: Registry for rare tumors in children and adolescents http://www.klinikumdo.de/fs1/medizin/kliniken-und-abteilungen/klinik-fuer-kinder-und-jugendmedizin/	Germany
Austrian Cancer Registry http://www.statistik.at/web_de/statistiken/index.html	Austria
Estonian Cancer Registry http://www.tai.ee/en/r-and-d/registers/estonian-cancer-registry	Estonia
Suomen Syöpärekisteri (Finnish Cancer Registry) http://www.cancer.fi/syoparekisteri/en/	Finland
Epidemiological Cancer Registry Baden-Württemberg http://www.dkfz.de/de/krebsregister/index.html	Germany
Bayern Population Based Cancer Registry	Germany
MCR—Munich Cancer Registry http://www.tumorregister-muenchen.de/	Germany
Common Cancer Registry of Berlin, Brandenburg, Mecklenburg-Western Pomerania, Saxony-Anhalt, and the free states Saxony and Thuringia http://www.berlin.de/gkr/	Germany
Bremen Cancer Registry http://www.krebsregister.bremen.de/home.html	Germany
Hamburg Cancer Registry http://www.hamburg.de/krebsregister/	Germany
German Childhood Cancer Registry (Partner of ACCIS: Automated Childhood Cancer Information System) http://www.kinderkrebsregister.de/dkkr/ueber-uns/uebersicht.html	Germany
EKRS: Saarland Cancer Registry http://www.krebsregister.saarland.de/	Germany
German Central Children Tumor Registry http://www.patho.uni-kiel.de/	Germany
Schleswig-Holstein Cancer Registry http://www.krebsregister-sh.de/	Germany
Icelander Cancer Registry http://www.krabbameinsskra.is/indexen.jsp?id=b	Iceland
Maltese Cancer Registry http://ehealth.gov.mt/HealthPortal/strategy_policy/healthinfor_research/registries/cancers.aspx	Malta
Norwegian Cancer Registry https://www.kreftregisteret.no/en/	Norway
Kielce Cancer Registry https://www.onkol.kielce.pl/pl/onkol?destination=onkol	Poland
Cracow Cancer Registry http://www.orpha.net/consor/cgi-bin/ResearchTrials_RegistriesMaterials.php?lng=EN&data_id=76337&RegistryMaterialName=Mazowiecki-Rejestr-Nowotworow-nalezacy-do-projktu-RARECARE&title=Mazowiecki-Rejestr-Nowotworow-nalezacy-do-projktu-RARECARE&search=ResearchTrials_RegistriesMaterials_Simple	Poland
Mazovian Cancer Registry http://www.orpha.net/consor/cgi-bin/ResearchTrials_RegistriesMaterials.php?lng=EN&data_id=76337&RegistryMaterialName=Mazowiecki-Rejestr-Nowotworow-nalezacy-do-projktu-RARECARE&title=Mazowiecki-Rejestr-Nowotworow-nalezacy-do-projktu-RARECARE&search=ResearchTrials_RegistriesMaterials_Simple	Poland
Registo Oncológico Regional Sul http://www.ror-sul.org.pt/pt	Portugal
National Cancer Registry http://www.nczisk.sk/en/Pages/default.aspx	Slovakia
Slovenian Cancer Registry http://www.onko-i.si/	Slovenia
Swedish Childhood Cancer Registry http://ki.se/en/kbh/paediatric-oncology	Sweden

Finally, a number of institutions and laboratories, in close collaboration with neurosurgical teams, are pioneering the creation of LGG tumor banks. For example, the Pediatric Low-Grade Astrocytoma Program at the Dana Farber Cancer Institute has invested in a tissue banking research effort to abstract data of each tumor and distribute the frozen tissue to investigators. Similar biospecimen repositories also exist at Johns Hopkins University, St. Louis Children‘s Hospital/Washington University, Children‘s National Medical Center, and St. Jude Children‘s Research Hospital. Nevertheless, the general lack of frozen tissue samples has hindered comprehensive analyses of cooperating molecular changes in NF1-associated PA. The lack of surgical specimens reflects the current practice of treating these tumors without a tissue diagnosis because current clinical practices exclude giving patients a biopsy unless they present clear abnormal clinical or radiological symptoms. However, by pooling these rare biopsy materials from a large number of institutions, programs like the NF1 Synodos LGG Initiative, spearheaded by the Children‘s Tumor Foundation, aim to perform comprehensive genetic and genomic sequencing of these tumors.

### Cell Lines

Cell lines are typically derived from patients or animal models. Establishing cell lines from patient tumors would be ideal for testing preclinical drugs because these lines closely resemble actual human tumors and may more closely mirror the response of a particular patient‘s tumor to treatment. Unfortunately, despite efforts by many research groups, human NF1-PA cell cultures have yet to be successfully established. It is not clear whether this reflects issues with cellular senescence or the need for trophic growth factors from the normal microenvironment. As such, continual passage of sporadic tumors is associated with loss of the signature fusion BRAF alteration (*KIAA1549:BRAF*) and oncogene-induced senescence (35). To date, only a small number of PA cell lines and their genetic characteristics have been reported, which are summarized in Table 2, including NCH492 (*BRAF* duplication (4)), IPNT-H (36), and Res186 (*PTEN* deletion and *TP73* promoter hypermethylation (37, 38)). However, none of the LGG cell lines have reported *NF1* gene loss/mutation. Further characterization of the existing sporadic LGG cell lines as well as the development of NF1-LGG cell lines are needed.

**Table 2:** **Demographic and biological features of glioma cell lines**.

Name	Age	Gender	Histological subtype	Year made	Mutations	Primary PMID Ref(s)	Other Refs (mutations only)	Note(s)	Originating investigator/and institution or other source(s)
CHLA-03-AA	9 years	F	AA			25211508			Anat Erdreich-Epstein (Saban Research Institute, Children‘s Hospital Los Angeles); ATCC

IPNT-H	6 months	M	Hypothalamus PA		Positive GFAP, A2B5, and CD44. Weakly positive vimentin	9428346			Dr. Geoffrey Pilkington (Institute of Psychiatry, London, UK)

NCH134	Adult		DA	1996–2004		18398503			Dr. Christel Herold-Mende (Department of Neurosurgery, University of Heidelberg)

NCH480b	Adult		Oligoastrocytoma	1996–2004		18398503			Dr. Christel Herold-Mende (Department of Neurosurgery, University of Heidelberg)

NCH492	6 years	Mix	Nervous system; PA	1996–2004	Large copy number gain of chromosome arm 7q spanning the BRAF locus by array-CGH	18398503			Dr. Christel Herold-Mende (Department of Neurosurgery, University of Heidelberg; Sigma-Aldrich)

NCH514	Adult		Oligoastrocytoma	1996–2004		18398503			Dr. Christel Herold-Mende (Department of Neurosurgery, University of Heidelberg)

Res186			PA		Homozygous PTEN deletion at 10q23; active mTORC1/mTORC2; specific promoter hypermethylation TP73; high levels of phoso-Akt by WB; Phosphorylated GSK3β levels were low; S6 activation, methylated GSTP1	19365568	24203892	Suppressed by MK8669 (greater than Res259)	Dr. Michael Bobola (University of Washington, Seattle, WA, USA)

Res259	Pediatric	DA			PFGFRA gain, CDKN2A deletion; active mTORC1/mTORC2; specific promoter hypermethylation FHIT, HIC1; Phosphorylated GSK3β levels were low by WB, high pS6 by WB	19365568	24203892		Dr. Michael Bobola University of Washington, Seattle, WA, USA

SF188	Pediatric	GB			7% CD133 positive cells, disruption of p53 pathway via point mutation TP53, RTK/PI3K/AKT pathway via NF1 deletion				Dr. Daphne Haas-Kogan (UCSF, San Francisco, CA, USA),

UW479	Pediatric		AA		APC, CASP8, CD44, CDH13, CHFR, ESR1, GSTP1, IGSF4, MGMT, PAX5A, PAX6, RARB, methylated RASSF1A and TMS1, methylated GSTP1	19365568			Dr. Michael Bobola (University of Washington, Seattle, WA, USA)

*AA, anaplastic astrocytoma; DA, diffuse astrocytoma; GBM, glioblastoma multiforme; HGG, high-grade glioma; PA, pilocytic astrocytoma; GB, glioblastoma, WT, wild type*.

At the Washington University NF Center, investigators launched the NF1 Brain Trust Project in October 2012 to create a repository of NF1 patient-induced pluripotent stem cell (iPSC) lines. While these are not glioma cells, they provide a renewable source of human cells for cellular reprograming. Importantly, these iPSC lines were instrumental in demonstrating that different germline *NF1* gene mutations result in different levels of neurofibromin expression (39). Current work is underway to use these lines as cellular substrates for glioma modeling, as part of the CTF NF1 Synodos Initiative.

### Genetically Engineered Mouse Models

In the absence of patient-derived cell lines or xenografts, genetically engineered mice (GEM), listed in Table 3, have emerged as instructive models for elucidating the cellular and molecular determinants critical for glioma formation and growth. While mice with a germline *Nf1* gene mutation (*Nf1*+/− mice) do not develop gliomas, neither do mice in which both copies of the *Nf1* gene are inactivated in neuroglial progenitors (40), suggesting that bi-allelic *Nf1* gene inactivation in the proper cell of origin is not sufficient for glioma formation. To better model NF1-PA arising in children with a germline *NF1* gene mutation, *Nf1*+/− mice were generated in which *somatic* loss of the remaining functional *Nf1* allele occurs in neuroglial progenitors (40–42). Analysis of the resulting mice revealed that gliomas arise between 10 and 12 weeks of age in the prechiasmatic optic nerves and chiasm with >90% penetrance. While these LGGs lack the characteristic eosinophilic granule bodies and Rosenthal fibers commonly found in NF1-PA, the murine optic glioma tumors share several similarities with their human counterparts. These tumors display frank tissue distortion evident on MRI, low proliferative indices, increased microglia infiltration, and axonal damage, which culminate in retinal ganglion cell (RGC) loss and reduced visual acuity (40, 43–45).

**Table 3:** **Transgenic Mouse Models**.

Name	Penetrance, onset	Histological subtype	References (PMID)		
*FMPC*		Differentiated neurons, embryonal	25246427		
Nf1 (flox/mut); GFAP-Cre (FMC or Nf1+/–^GFAP^CKO)	100%, 2 months	Differentiated neurons, embryonal; neuroglial	12077339	25772366	Used Cre/LoxP technology
Nf1 (flox/flox); GFAP-Cre		Differentiated neurons	14695164		Poor breeders
Nf1 (flox/mut)		Embryonal, Neurofibromin			GFAP-Cre line 73.12 mice (Stock No. 012886)
Nf1 (flox/flox)		Embryonal, Neurofibromin	11297510		
Nf1+/–		Embryonal, Neurofibromin	7920653		CCE-ES cells (45) 10f and 38a clones
*Nf1*+/–^KRas^	100%, 2 months	Mixed embryonal and lung adenocarcinoma			
			11751630		
*Nf1*+/–^GFAP-m^CKO	20%, 4 months	Embryonal			
NF1^Syn1^KO					No tumor growth

Recent studies have also discovered co-existing genetic changes in NF1-PA in addition to *NF1* gene mutation, including heterozygous *PTEN* deletion and *KIAA1549:BRAF* duplication (46). Based on these observations, two *Nf1* optic glioma GEM models were generated that additionally harbored either a heterozygous *Pten* deletion or *KIAA1549:BRAF* overexpression. While *KIAA1549:BRAF* overexpression did not provide an additional growth advantage, tumors from mice with a co-existing heterozygous *Pten* deletion had larger volumes, increased proliferation indices, and more microglia infiltration. Furthermore, inhibition of *Nf1*+/− non-neoplastic stromal cells (microglia) significantly decreases optic glioma growth and maintenance (47). These preclinical mouse models suggest that additional genetic changes in NF1-associated PA patients differentially influence tumor growth relevant to the design of future therapeutic strategies.

### Xenografts and Cancer Stem Cells

Whereas xenografts of human tumors maintained in immunocompromised mice have been regularly employed to predict the efficacy of candidate drugs, most of these xenografts derive from high-grade (malignant) cancers. In contrast, generating neoplastic cells from PA tumors has been challenging due to their low clonogenic nature and the requirement for a permissive tumor microenvironment. As presented in Table 4, only a few patient-derived xenograft (PDX) models of sporadic PA have been reported. Two such models, BT-35 and BT-40, have been used in a preclinical study of the selumetinib MEK inhibitor (48). BT-35 and BT-40 express WT and mutant *BRAF* (*BRAF^V600E^*; constitutively active *BRAF*), respectively. While BT-35 xenografts continued to grow in the presence of selumetinib, BT-40 xenografts are highly sensitive to MEK inhibition. To date, no PDX models exist for NF1-PA.

**Table 4:** **Xenografts**.

	Name	Histological subtype	Mutation	Reference (PMID)
Subcutaneous	BT-35	PA/AA	WT BRAF	20806365
	CB17SC-M scid−/− female mice with BT-40 tumor	PA/atypical teratoid malignant rhabdoid	Mutant [V600E] BRAF	20806365

To develop robust human HGG PDX models, several groups have derived cancer stem cells (CSCs) as *in vitro* and transplantable *in vivo* platforms for drug discovery and evaluation (49–51). Similar to their non-neoplastic counterparts, CSCs are capable of self-renewal and multilineage differentiation (52–54). Unfortunately, the CSCs generated from PA tumors have clonogenic frequencies between 0.3 and 1.5% and often undergo oncogene-induced senescence (55). As such, no PA CSC models have been developed from human specimens.

Using GEM, the process of establishing and maintaining human NF1-PA CSC xenografts can be optimized. Using *Nf1* optic glioma GEM models, LGG CSCs have been isolated, which were capable of self-renewal, long-term passage, and multilineage differentiation (56). Importantly, these CSCs formed LGG-like lesions when injected into the brainstems of immunocompetent, but not immunocompromised (athymic), mice. This latter finding underscores the obligate role of the tumor microenvironment in *Nf1* murine low-grade gliomagenesis. Further work using these CSCs may reveal the critical components of the tumor microenvironment required for CSC engraftment, which might be accurately recapitulated in rodents to enable xenografting of human NF1-PA tumors in the future.

## Potential Drug Targets

### Molecular Signaling Pathways

**Figure 2:**
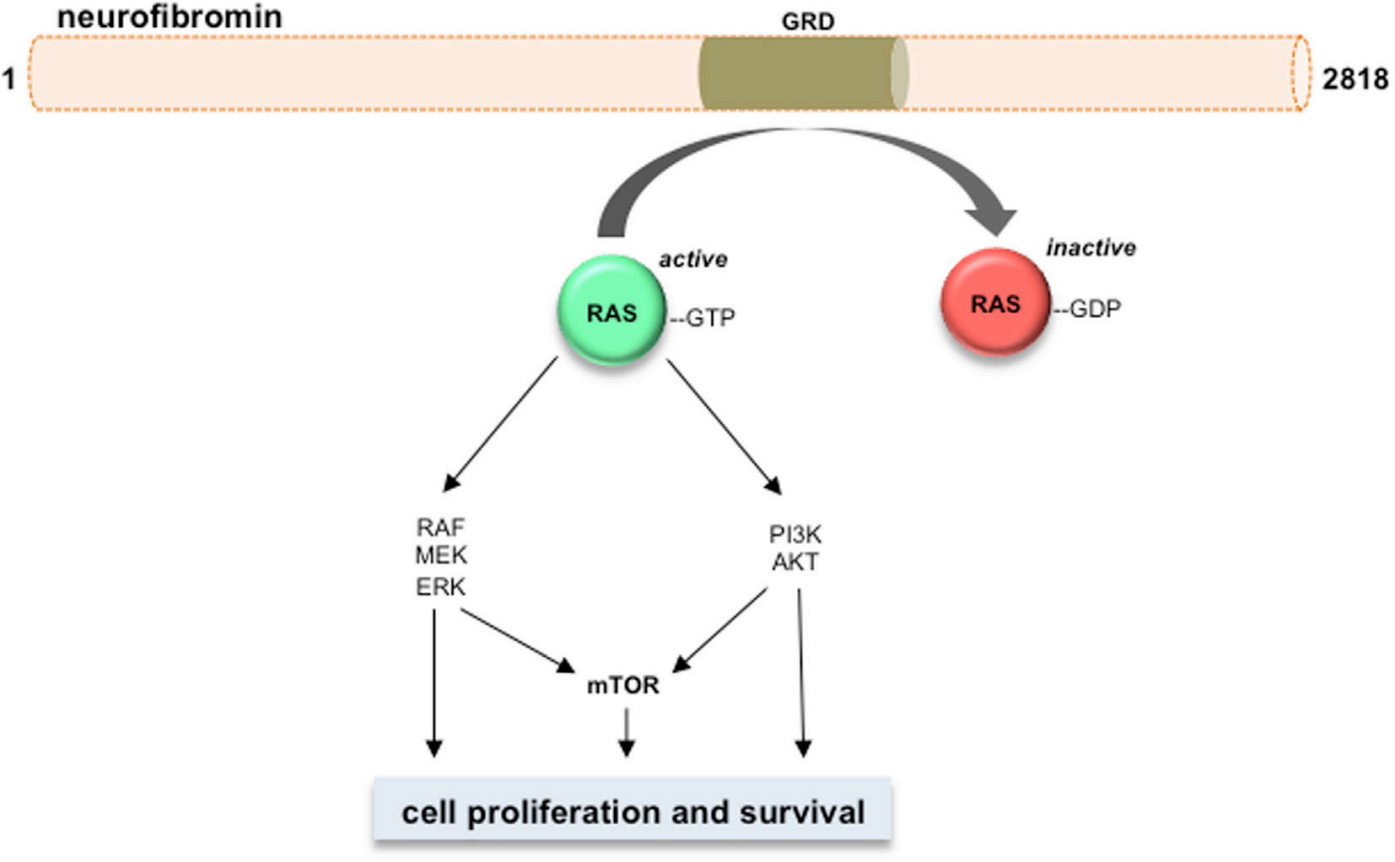
**Neurofibromin is a negative regulator of RAS and its downstream effectors**. Neurofibromin protein (2818 amino acids) contains a GAP-related domain (GRD) domain, which functions to inhibit RAS activity through accelerating RAS-GTP to RAS-GDP hydrolysis. The RAS downstream effector pathways are shown, including RAF/MEK/ERK, PI3K/AKT, and mTOR. Activation of these pathways increases cell growth and/or cell survival in *Nf1*-deficient neoplastic cells.

Neurofibromin functions primarily as a suppressor of RAS and its downstream signaling pathways (Figure 2) (57–62). Neurofibromin contains a GAP (GTPase-activating protein)-related domain (GRD) that accelerates the GTP hydrolysis of RAS, leading to RAS inactivation (63). PI3K/AKT and RAF/MEK/ERK are two common pathways downstream of RAS. Hyperactivation of the PI3K/AKT and MEK/ERK pathways are observed in *Nf1*-deficient neural stem cells (NSCs) (64) and astrocytes (65). While each RAS effector pathway has different functions in NSCs relative to proliferation and glial differentiation, both the PI3K/AKT and MEK/ERK pathways are hyperactivated in *Nf1*−/− astrocytes relative to their wild-type counterparts (62, 66–69). Importantly, these downstream effectors converge on the mTOR complex to regulate astrocyte growth *in vitro* and *Nf1* optic glioma proliferation *in vivo* (9). Future laboratory investigations will be required to define the RAS effector signaling networks required for optic glioma maintenance relevant to adaptive and potential escape mechanisms from targeted therapies that might be operative in these tumors (56).

### Tumor Microenvironment

**Figure 3:**
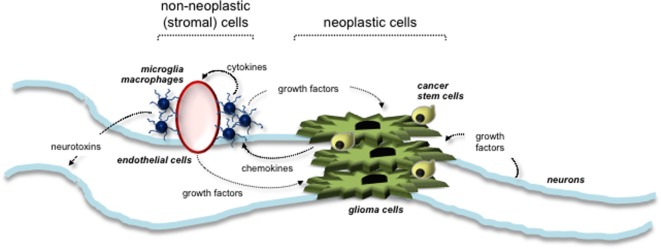
**Tumor microenvironment stimulates the growth of NF1-associated PA**. Interactions between neoplastic cells (glioma cells and cancer stem cells) and non-neoplastic (stromal) cells are depicted. Stromal cells within the tumor environment include neurons, microglia, macrophages, and endothelial cells. Arrows indicate the paracrine factors (e.g., chemokines and growth factors) secreted by each cell type and their responsive cells.

Evidence from several different experimental studies has revealed a critical role for the tumor microenvironment in murine *Nf1* optic glioma formation and growth. Similar to other LGG systems, the tumor microenvironment of *Nf1* optic glioma consists of neoplastic cells (glioma cells and CSCs) and non-neoplastic stromal cells (neurons, endothelial cells, and monocytes) (Figure 3). Increasing evidence suggests that neoplastic cells secrete paracrine factors (e.g., chemokines) to recruit stromal cells and stimulate growth factor/cytokine production from these cells. The growth factors then interact with their cognate receptors on the cell surface of neoplastic cells to increase cell proliferation. The roles and interactions of these cells within the microenvironment of NF1-PA remain to be completely elucidated. To date, the importance of microglia to PA pathogenesis is underscored by the finding that 35–50% of the cells in these low-grade tumors are Iba1^+^ tissue macrophages (monocytes) (70). Monocytes in the brain can represent either resident tissue macrophages (microglia) that enter the brain during mid-embryonic development or peripheral macrophages from the bone marrow (71). In mouse *Nf1* optic gliomas, the monocytes are Cd11b^+^, Cd45^low^, Cx3cr1^+^ microglia (70). These microglia are critical for gliomagenesis, such that impairing their directional migration into the tumor by genetic reduction of the Cx3cl1 receptor (Cx3cr1) delays optic glioma formation (72). Moreover, pharmacologic inhibition of microglia function with minocycline (73) or c-Jun-NH2-kinase (JNK) inhibitors (74) reduces optic glioma proliferation. In addition, genetic elimination of microglia using transgenic mice expressing the *CD11b-thymidine kinase* (*TK*) transgene results in dramatic reduction in optic glioma proliferation (70). Collectively, these observations establish that microglia in the tumor microenvironment produce critical growth factors that increase optic glioma growth.

To identify potential growth factors elaborated by glioma-associated microglia, RNA-sequencing of murine optic glioma-associated microglia has revealed that the Ccl5 chemokine produced by tumor-associated microglia is a potent stromal factor important for optic glioma maintenance in mice (47). In this regard, Ccl5 was sufficient to increase *Nf1*-deficient astrocytes growth *in vitro*, and its inhibition using neutralizing antibodies reduced optic glioma proliferation *in vivo*. Current studies are underway to identify additional chemokines and growth factors for potential stroma-directed glioma therapies.

### Visual Outcome

While 15–20% of NF1 patients develop OPGs, 30–50% will experience impaired visual acuity. Since surgery is rarely employed and radiation is associated with the development of secondary malignancy (75, 76), the standard of care entails the use of chemotherapy. One of the applications of mouse *Nf1* optic glioma models is to define the mechanisms underlying tumor-associated visual decline. As such, *Nf1* optic glioma mice develop a time-dependent sequence of events, beginning with axonal injury at the tumor site, followed by axonal degeneration, RGC loss (apoptosis), retinal nerve fiber layer thinning, and reduced visual acuity (44, 77).

Neurofibromin plays critical roles in protecting neurons from degeneration and cell death. As such, RGCs harboring a germline *Nf1* gene mutation (*Nf1*+/−) have smaller growth cone areas, shorter neurite lengths, and increased cell death compared to wild-type RGCs (78). These abnormal cellular phenotypes are due to impaired neurofibromin regulation of cyclic AMP (cAMP) generation. In these neurons, reduced neurofibromin expression results in lower cAMP levels, such that elevating cAMP levels using pharmacological methods increases the growth cone areas and neurite lengths of *Nf1*+/− RGCs *in vitro*. In addition, elevating cAMP levels greatly attenuates RGC cell death in *Nf1* optic glioma mice *in vivo* (78). These observations raise the potential of future neuroprotective strategies aimed at reducing RGC death.

### Current Pediatric NF1-LGG Clinical Trials

With respect to NF1-LGG, selumetinib, a MEK inhibitor that inhibits the growth of a BRAF^V600E^-expressing PA xenograft (48) and blocks growth in both *Nf1* optic glioma (9) and *KIAA1549:BRAF*-driven NSC glioma (11) models, has been evaluated in a phase I clinical trial (http://clinicaltrials.org #NCT01089101) for pediatric LGG. Since the PI3K/AKT and MEK/ERK pathways converge on mTOR, treatments that target mTOR may still have utility. Several mTOR inhibitors other than sirolimus have been investigated for treating NF1- and sporadic LGG. Everolimus (RAD001), a rapamycin analog that targets mTOR, is currently being explored as a potential treatment in two separate trials (http://clinicaltrials.org #NCT01158651 and #NCT01734512, recruiting).

## Future Models and Developing New Therapeutic Drugs

### Increase Biobanking and Database Efforts

Especially relevant to NF1-LGG, proper communication and collaboration among patient families, clinicians, and researchers are highly recommended in order to preserve valuable LGG specimens for molecular characterization. The importance of donating tumor samples for research purposes should be emphasized to medical groups and families – especially for patients affected by NF1-PA, for which surgical biopsy samples are sometimes sparingly small. Legacy gifts of autopsy tumor tissue and selfless donation can similarly propel the field forward, while simultaneously providing hope for families for whom treatments are not currently available. Most pediatric cancer families (93%) would agree to autopsies if given the option (79); however, there has been a decrease over the past several decades in the acquisition of autopsy specimens for research (79). Patients, advocates of patients, clinicians, and researchers should aim to spread awareness, enhance subject recruitment, and increase funding for the future storage of cryo-viable frozen tissue samples at one or multiple sites, with cataloging by way of a HIPAA-compliant online database such as the CTF NF1 Registry.

### Expand the Spectrum of Nf1 GEM Models

While *Nf1* mutant mouse models provide unprecedented opportunities for drug discovery and evaluation, the current collection of these strains does not fully capture the full spectrum of human clinical heterogeneity. Laboratory investigations have begun to identify the various factors that might underlie this inherent clinical heterogeneity. First, genotype–phenotype correlation studies in people with NF1 have revealed intriguing associations (80–84); the most striking of which is the finding that individuals with p1809 codon mutations do not develop dermal neurofibromas (85). Relevant to brain tumors, NF1 patients with 5′ *NF1* gene mutations have higher chance developing optic gliomas compared to patients with mutations at other locations of the *NF1* gene (82). Based on these types of clinical observations, human iPSCs have been used to demonstrate that different *NF1* mutations lead to different levels of neurofibromin protein loss (39, 86, 87). These results suggest that not all *NF1* gene mutations are equivalent.

To explore this further, GEM have recently been engineered to harbor specific *Nf1* mutations seen in people with NF1. This is in contrast to the current *Nf1* optic glioma mouse model in which the *Nf1* gene is inactivated by the insertion of a neomycin targeting cassette, a genetic alteration not seen in individuals with NF1 (88, 89). Strikingly, in these proof-of-principle studies, *Nf1* mice harboring a representative missense mutation did not develop optic gliomas, whereas those with a representative nonsense mutation harbored optic gliomas with higher proliferative indices than the conventional *Nf1* optic glioma strain (90). Future preclinical studies should be designed to incorporate mice with different germline *Nf1* gene mutations and cooperating genetic changes (heterozygous *Pten* loss; *KIAA1549:BRAF* expression) as a way of more accurately representing the full spectrum of disease in this patient population.

### Optimize Patient-Derived Xenograft Methodologies

As mentioned above, the growth of LGG PDXs likely requires trophic support from the tumor microenvironment. Current xenograft methodologies developed using HGG cells may not be suitable for generating LGG PDX due to the relative stromal independence of HGG cells. The methodologies for future PA PDX studies can be tested and optimized using xenografts developed from mouse NF1-LGG cells (56). Current mouse xenograft methodologies involve the isolation of single cells (CD133+) from the optic nerves of 3-month-old mice with NF1-LGG (*Nf1*+/−^GFAP^CKO mice) (56). The resulting glioma stem cells express several stem cell markers and a response to therapeutic treatments similar to that observed in humans. This model lays the groundwork for further investigation into potential PDXs.

## Summary

Since the discovery of the *NF1* gene in 1990, great advances have been made to understand the cellular and molecular mechanisms underlying NF1-PA pathogenesis and maintenance. With the establishment of biobanking and registry initiatives, NF1-LGG specimens can be better characterized for actionable mutations. The resulting mutations and genomic alterations can next be evaluated in preclinical model systems as a means of identifying their contributions to tumor growth and targets for therapeutic intervention. Moreover, the incorporation of these cooperating genetic/genomic changes in combination with other factors, like the germline *NF1* gene mutation, create a more representative spectrum of models that capture the innate clinical heterogeneity of these tumors for preclinical drug assessment and biomarker discovery. The implementation of these strategies offers the greatest opportunities to discover effective treatments for these common brain tumors, both for children with NF1 and those with non-syndromic LGG.

## Author Contributions

All authors designed the study and wrote the manuscript.

## Conflict of Interest Statement

The authors declare that the research was conducted in the absence of any commercial or financial relationships that could be construed as a potential conflict of interest.

## Abbreviations

cAMP, cyclic adenosine monophosphate; CSC, cancer stem cell; ERK, extracellular signal-regulated kinase; GAP, GTPase-activating protein; GEM, genetically engineered mice; GRD, GAP-related domain; HGG, high-grade glioma; iPSC, induced pluripotent stem cell; JNK, c-Jun-NH2-kinase; PA, pilocytic astrocytoma; LGG, low-grade glioma; MAPK, mitogen-activating protein kinase; MEK, mitogen-activated protein kinase kinase; mTOR, mechanistic target of rapamycin; NF1, neurofibromatosis type 1; NSCs, neural stem cells; PDX, patient-derived xenograft; PI3K, phosphoinositide 3-kinase; PKCγ, protein kinase C gamma; RAF, rapidly accelerated fibrosarcoma; RAS, rat sarcoma; RGC, retinal ganglion cells.
